# Baleen reveals sex-specific foraging, reproduction and pregnancy endocrinology in sei whales

**DOI:** 10.1098/rsos.261471

**Published:** 2026-10-28

**Authors:** Daniela Magalhaes Drummond de Mello, Alejandro A. Fernandez Ajó, Lilian Reiss, Kathleen Hunt, Matthew C. Rogers, Danielle Dillon, Faith M. Walker, Anna Riley, Daniel E. Sanchez, Christoph Mayr, Vreni Häussermann, Keri-Lee Pashuk, Matthew R. Coyle, Fernando Ribeiro Gomes, C. Loren Buck

**Affiliations:** ^1^ Department of Physiology, Institute of Biosciences, University of São Paulo, São Paulo, Brazil; ^2^ Department of Biological Sciences, Northern Arizona University, Flagstaff, AZ, USA; ^3^ School of Forestry and the Pathogen and Microbiome Institute, Northern Arizona University, Flagstaff, AZ, USA; ^4^ Fisheries, Wildlife, and Conservation, Hatfield Marine Science Center Oregon State University, Newport, OR, USA; ^5^ Institute of Geography, Friedrich-Alexander-Universität Erlangen-Nürnberg, Erlangen, Germany; ^6^ CNRS-Museum National d'Histoire Naturelle, Paris, France; ^7^ Marine Mammal Institute, Oregon State University, Corvallis, OR, USA; ^8^ National Oceanic and Atmospheric Administration, Juneau, AK, USA; ^9^ Anderson Cabot Center for Ocean Life, New England Aquarium, Boston, MA, USA; ^10^ Facultad de Recursos Naturales, Universidad Católica de Valparaíso, Valparaíso, Chile; ^11^ Patagonia Projects, Puerto Éden, Chile; ^12^ Department of Community, Environment and Policy, The University of Arizona Mel and Enid Zuckerman College of Public Health, Tucson, AZ, USA

**Keywords:** *Balaenoptera borealis*, mass stranding, stable isotopes, baleen growth rate, niche width, gestation length, progesterone, glucocorticoids, reproductive cycles

## Abstract

The largest baleen whale mortality event on record, when more than 340 sei whales (*Balaenoptera borealis*) stranded in Chilean Patagonia during the extreme 2015 El Niño, provided a unique opportunity to reconstruct multi-year individual life histories from baleen plates. Baleens of seven females and four males were sampled to examine sex-specific foraging strategies, reproductive cycles and endocrine patterns through integrated molecular, isotopic and hormonal analyses. Sex was identified using a refined DNA amplification protocol. Patterns of *δ*^13^C and *δ*^15^N revealed significant annual oscillations, enabling estimates of baleen growth rates (15.7–20.5 cm yr^−1^) and retrospective timelines of up to 3.5 years. Males exhibited broader isotopic niche widths, suggesting sex-related foraging differences. Progesterone was detected in all samples, with sustained elevations in females indicating pregnancies of 7.7–10.7 months. One female exhibited two complete pregnancies recorded in baleen, enabling estimation of a 1.3-year intercalving interval. Concurrent elevations of cortisol and corticosterone were observed during pregnancy. Conceptions and births were distributed across multiple seasons, with no clear evidence of seasonal concentration. Together, these findings provide the first baleen-based reconstruction of sei whale reproductive history and endocrine variation, establishing critical baselines for assessing population resilience and informing conservation under rapid environmental change.

## 1. Introduction

During the late austral summer and early autumn of 2015, a mass mortality event occurred along the Chilean coast, involving at least 340 stranded sei whales (*Balaenoptera borealis*). This event constitutes the largest documented mass mortality of baleen whales to date [1]. The stranding event occurred in the central area of Chilean Patagonia, specifically around the Taitao Peninsula and Golfo de Penas [1]. The exact date of death for individuals is unknown, given the area's remoteness; however, it was estimated to have occurred between mid-February and mid-April 2015 based on drift model parameters [1]. Cetacean strandings in Chilean Patagonia are not uncommon and have been recorded since 1968, comprising 35 different species of whales and dolphins [2]. However, the number of sei whales washed ashore in this event was exceptionally high, possibly linked to acute mortality from a harmful algal bloom (HAB) exposure during an atypically strong El Niño–Southern Oscillation (ENSO) in that year [1]. Because HABs typically cause acute mortality, it is likely that these whales were relatively healthy and exhibited normal physiology prior to their deaths, thereby providing baseline physiological and endocrine parameters.

Collecting samples from dead-stranded cetaceans allows the acquisition of tissues for analysis of biomarkers of physiology, endocrinology and ecology of whales, which can be logistically challenging and/or costly to assess by other means in free-living cetaceans. The feeding ecology, diet preferences and relative proportions of prey types of whales can be estimated through the analysis of stable isotope ratios of carbon (*δ*^13^C) and nitrogen (*δ*^15^N) along baleen plates in a retrospective temporal scale [3,4]. Physiological processes related to metabolism, response to stressors and reproduction of whales can be investigated by extracting and quantifying hormones that accumulate in baleen and several other sample types such as blubber, faeces and earplugs [5–10]. Among these, earplugs and baleen are the only sample types that allow for a retrospective time-series analysis, with baleen providing fewer years of information but superior temporal resolution (e.g. summer versus winter and duration of gestation).

Baleen is the filter-feeding apparatus of mysticete whales, composed of long, narrow plates of keratinized tissue that are firm, flexible and resistant to degradation [11]. Therefore, studies involving archival baleen, or detached plates from the skull collected directly on the shore, can yield reliable data, even if the collection occurred years after the animal's death. Some molecules, like hormones, contaminants and stable isotopes, are relatively stable in this tissue type for decades [12–14]. However, DNA amplification from baleen is not always successful owing to low DNA quality/quantity, hindering specific analyses such as sex determination of individuals, a crucial piece of information when studying basic biological parameters. Molecular sex determination from baleen has previously been demonstrated in archaeological baleen whale material [15]. To date, hormone analyses from baleen have accessed information regarding the sex of individuals through direct observation of the genital slit after stranding or subsistence hunt, observation of gonads during necropsies or historical records of known individuals in the wild [6,16,17]. In some cases, however, the sex of the individual is not known or was not recorded, and hence a genetic method to determine sex from baleen would be highly useful. Protocols for extracting and amplifying ancient or degraded DNA [18–20] may prove helpful but have not yet been investigated for baleen. One alternative to circumvent this problem would be to optimize DNA amplification protocols, particularly for quantitative PCR (qPCR), which is more sensitive than traditional endpoint PCR. Thus, one goal of our study was to evaluate DNA amplification protocols for application to sex determination from sei whale baleen.

Baleen grows continuously from the upper jaw while eroding at the distal tip during feeding, creating a chronological record in which stable isotopes, hormones and other circulating biomarkers are incorporated into the keratin matrix over time [3,13,14,21–23]. Data from a particular point along a baleen plate can thus be assigned to a specific calendar year and month of the whale's last several years of life, provided that the (species- and age-specific) baleen growth rate (BGR) and date of death are known or can be estimated. BGR can be determined via *δ*^13^C and *δ*^15^N, which, in seasonally migrating mysticetes, typically display repeating oscillations (stable isotope cycles) along the baleen, thought to reflect seasonal changes in foraging along the migration route (e.g. summer versus winter) [3,24,25].

Once the BGR has been determined for a given plate of baleen, analysis of hormones along the length of the baleen allows study of reproductive cycles of these long-lived, migratory organisms. This information is fundamental to understanding the timing and plasticity of reproduction and forecasting population resilience in the face of environmental change. For example, reproductive seasonality, as well as the timing of the breeding season and the location of potential breeding grounds, has been deduced from the annual fluctuations of baleen testosterone in male mysticete whales (bowhead whale *Balaena mysticetus*, North Atlantic right whale *Eubalaena glacialis* and blue whale *Balaenoptera musculus* [26,27]). Additionally, marked and sustained elevations of progesterone in baleen of females coincide with known pregnancies, as confirmed by calf sightings or necropsy (bowhead, North Atlantic right whale and blue whale [9,13]), providing critical biological validation for this technique. The interval between successive peaks in progesterone can then be used to estimate reproductive parameters such as intercalving interval, variability in that interval, frequency of calf loss, age of sexual maturity and potential impacts on population vital rates.

Glucocorticoid concentrations may increase in response to stressor stimuli and have been documented in the baleen of a bowhead whale and a humpback whale (*Megaptera novaeangliae*) that died following entanglement in fishing gear [8,28], nutritionally stressed and chronically ill humpback whales [8], and southern right whales exposed to seagull microparasitism [29,30]. Baleen glucocorticoid profiles in these studies were consistent with the expected adrenal stress response, showing gradual, dose-dependent elevations with increasing intensity of stressor exposure. However, glucocorticoid elevations are not exclusively associated with stress. Increased corticosterone concentrations have also been documented during pregnancy in bowhead and North Atlantic right whales, particularly during the second half of gestation [17,31]. Understanding glucocorticoid variation associated with normal reproductive processes is therefore essential for distinguishing endocrine changes related to physiological state from those resulting from environmental or anthropogenic stressors.

The sei whale is the third-largest rorqual and was heavily exploited during commercial whaling, particularly in the Southern Hemisphere between 1960 and 1972 [32]. Despite its cosmopolitan distribution, fundamental aspects of its reproductive biology and endocrinology remain poorly understood [33], largely owing to its elusive behaviour, offshore distribution and extensive seasonal migrations [34]. Most knowledge of sei whale reproduction derives from whaling records, which provide only point-in-time observations and do not allow longitudinal assessments of individual reproductive histories. Consequently, key parameters, such as gestation length and intercalving intervals (interval between the birth of successive calves, i.e. non-pregnant interval plus one gestation), remain uncertain. For example, gestation length has been estimated at 10.7 months based on fetal lengths [35], but no study has tracked pregnancy from beginning to end in an individual sei whale.

Pregnancy rate, defined as the proportion of sexually mature females carrying a fetus during mid- to late pregnancy, has been estimated for sei whales at 0.40–0.44 from whaling records [35] and 0.37 from necropsy data combining anatomy, histology and hormone analyses [36]. These values suggest an intercalving interval of about 3 years. However, no long-term studies have been conducted on any free-living sei whale population, leaving the accuracy of these estimates uncertain. Although some stable isotope data exist from sei whale baleen, hormone analyses have so far been limited to analytical validations confirming hormone detectability rather than providing biological and life-history insights [37]. As a result, fundamental aspects of sei whale endocrinology and reproduction remain poorly understood.

The present study aims to fill these knowledge gaps on the reproductive biology of sei whales by combining molecular techniques, stable isotope analysis and steroid hormone assessments from the baleen of sei whales stranded on the Chilean Patagonian coast. Our study has five goals: (i) refine a qPCR method using DNA extracted from baleen to accurately determine the sex of sei whales from baleen tissue samples. (ii) Advance knowledge of sei whale foraging ecology by assessing isotopic variation in baleen plates and by estimating the isotopic niche widths of males and females. Based on prior literature, we expect sex-specific differences in resource use or habitat to be detectable via baleen subsamples. (iii) Investigate pregnancy patterns and duration, as well as intercalving intervals, using cycles of *δ*^13^C and *δ*^15^N to determine the BGR together with progesterone concentrations. We expect progesterone in adult females to show periods with sustained elevations along the baleen corresponding to presumed pregnancies. Two complete pregnancies within the same baleen plate are expected to enable estimation of the calving interval. (iv) Investigate how glucocorticoid concentrations change across pregnancy and evaluate whether gestational elevations can serve as biomarkers of successful pregnancy. We predict a rise in glucocorticoid levels towards the end of pregnancy, consistent with the physiological changes commonly associated with mammalian parturition and lactation. (v) Assess migration phenology and reproduction timing using cycles of *δ*^13^C and *δ*^15^N. We anticipate finding evidence of reproductive synchrony within the population, with timing for conception and calving events predominantly occurring during the winter and spring seasons.

## 2. Material and methods

### 2.1. Baleen sample collection and pulverization

Baleen plates were collected in 2016 from a remote area around Golfo Tres Montes and Taitao Peninsula, Chile, between 47°S and 48°S in the Central Patagonian Zone, 1 year after the mass mortality event. Eleven baleen plates were collected from different carcasses on shore (one plate per individual), either by extracting loose baleen from skeletons or collecting loose plates in the sand, prioritizing the longest plate available from each individual (figure 1). All individuals with baleen plates ≥40 cm were classified as adults (*n* = 10) [38]. Two subadult/juveniles had 32 and 38 cm plates. All samples were dry and free of gingival tissue at the time of collection. Samples were transported from Chile to Northern Arizona University, USA, for processing and analysis. The baleen plates are currently housed at the University of Arizona and are appropriately identified according to the identification scheme described in the supplementary material.

**Figure 1.**
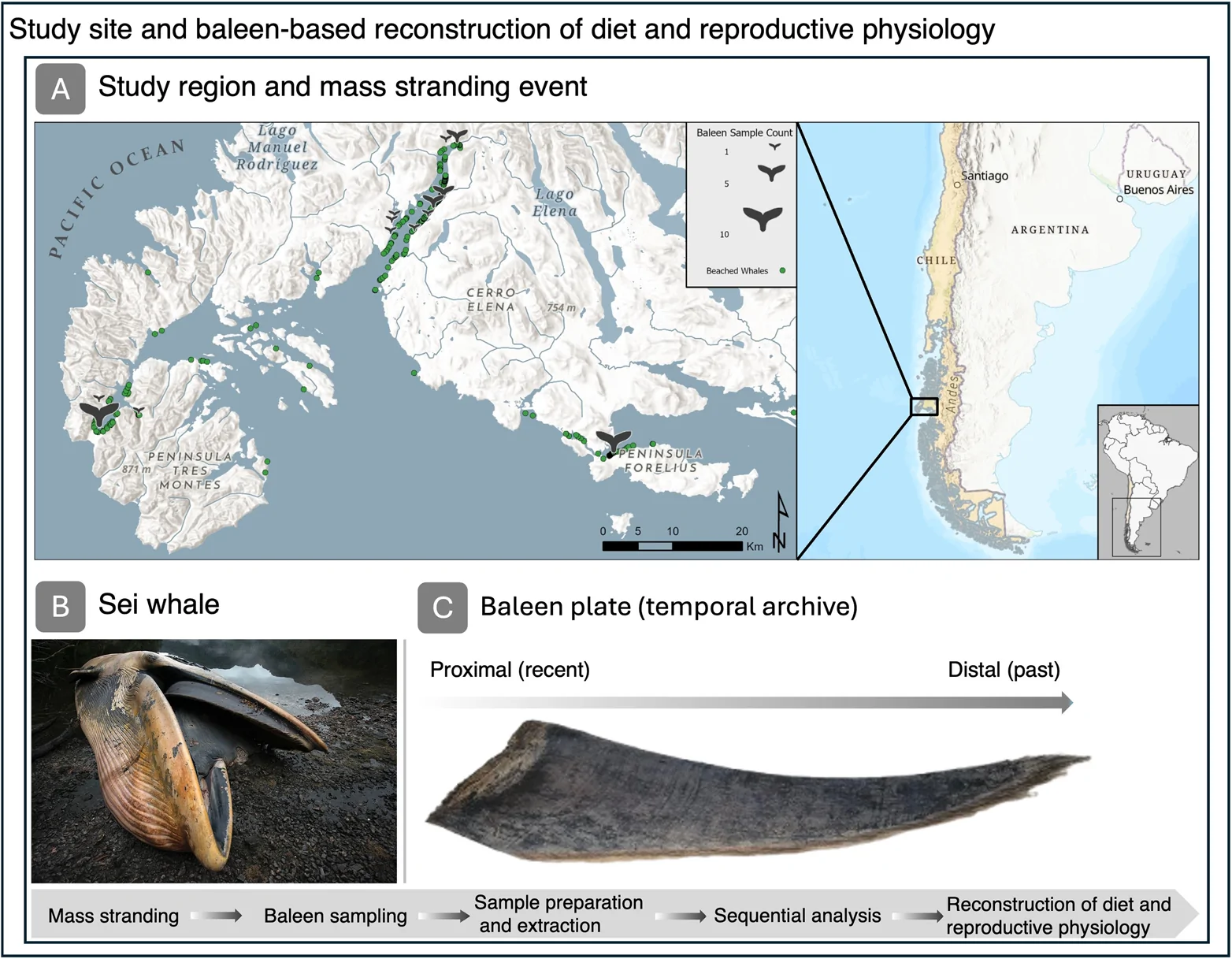
Study site and baleen-based reconstruction of diet and reproductive physiology. (A) Study region and location of the mass stranding event along the Central Patagonian Zone, Chile (47–48°S), including the Golfo Tres Montes and Taitao Peninsula. Map created in ArcGIS Pro (Esri Inc., Redlands, CA, USA). Basemap data sources include Esri, NASA, NGA, USGS, TomTom, Garmin, FAO and NOAA. (B) Example of a stranded sei whale during the 2015–2016 ENSO event. © Keri-Lee Pashuk. (C) Baleen plate illustrating its role as a temporal archive, with the proximal end representing the most recent growth and the distal end representing older growth.

Briefly, plates were cleaned with 70% ethanol, measured and affixed with tape along the length of the baleen marked at 1 cm intervals to guide subsampling [13]. Subsamples of baleen powder were collected every 1 cm along the labial edge of each plate using a handheld electric rotary grinder (Dremel model 395, type 5) fitted with a flexible extension equipped with a tungsten carbide ball tip. Sampling spanned the length of each plate from the proximal-most part that is embedded inside the gum tissue (0 cm point) to the distal tip, with sampling at each 1 cm point producing a shallow transverse groove. From each groove, around 80 mg of abraded baleen powder was collected for hormone and stable isotope analyses, weighed to the nearest 0.1 mg (Ohaus Explorer Pro EP214C analytical balance; Ohaus, Pine Brook, NJ, USA) and transferred to a borosilicate glass tube. A workstation ionizer (SPI No. 94000, SPI-wesstek.com) was positioned close to the balance to mitigate the potential effects of static electric charge on apparent sample mass [39]. Special care was taken to avoid cross-contamination between adjacent sampling points, by shielding other regions of the baleen with adhesive tape (VWR), thoroughly cleaning the entire plate with compressed air and a 70% ethanol wipe after each sample was collected and performing all sampling in a fume hood. In total, 580 powder subsamples were collected from the 11 baleen plates, which ranged from 32 to 67 cm in length. Stable isotope and hormone analyses were each performed on a subset of samples. Stable isotope subsampling was conducted at intervals of either 1 or 2 cm, while the first round of progesterone and glucocorticoid assays used a 4 cm subsampling interval. After identifying ‘high-progesterone regions’ indicative of potential pregnancies in each baleen plate, these regions were resampled for hormones at 1 cm intervals to improve temporal resolution (e.g. to resolve the timing of starts and ends of pregnancies). In total, of the 580 subsamples, 391 were used for stable isotope analyses in all 11 individuals, and 268 were used for hormone analyses in 9 individuals.

### 2.2. Sex identification

Since no visual identification of the sex was possible during sample collection at the beach, genetic sex identification was attempted for every baleen plate, using approximately 80 mg of powdered baleen collected from the very proximal edge of the baleen. These samples were extracted in a separate facility dedicated to the extraction of DNA from ancient and low-quality samples. Supplies for DNA sampling were decontaminated with UV exposure for either a 7 min or a 2 min contact period with 10% bleach, followed by 70% ethanol. We extracted DNA using a phenol–chloroform protocol developed for ancient samples [40]. The samples were centrifuged (Eppendorf 5810R) in Amicon filters (MilliporeSigma, Burlington, MA, USA) at 1461 × g in two 9-min cycles. Samples had final DNA volumes of 150 µl. To identify any contamination between samples, eight DNA extraction (reagent) blanks were extracted and prepared alongside the samples. Sample concentrations were measured using a Qubit 4.0 Fluorometer with the dsDNA High Sensitivity Kit (Invitrogen, Waltham, MA, USA) and standardized to 4 ng µl^−1^ with molecular grade water for screening.

We then screened each sample with primers and probes from [41] to target the ZFY gene of cetaceans. We prepared all PCR reagents in a ‘DNA-free’ room and loaded samples in a dedicated pre-amplification section of the laboratory within a decontaminated, contained workspace. We decontaminated the workspace, pipettes, and consumables using UV light, DNA AWAY™ (Fisher Scientific, Waltham, MA, USA) and 70% ethanol. We used only pre-sterilized, filtered pipette tips throughout the process [42,43]. For each screening target, we included PCR non-template controls, with molecular grade water substituted for DNA, and PCR positive controls of both known male and female DNA from North Atlantic right whales (*E. glacialis*), another mysticete species from which known-sex baleen was available (i.e. sex confirmed from necropsy data and individual sighting histories). Each reaction totalled 25 µl and included 12.50 µl of 2× TaqMan Environmental Master Mix 2.0 (Fisher Scientific, Waltham, MA, USA), 0.6 µM each primer, 0.1 µM each probe, 5 µl template DNA and molecular grade water making up the remaining volume. Each sample was screened in triplicate and run on a QuantStudio 7 Pro Real-Time PCR System (Applied Biosystems, Thermo Fisher Scientific, Waltham, MA, USA). PCR cycling conditions included a polymerase activation step of 94°C for 10 min, followed by 50 cycles of denaturation for 45 s at 94°C and combined annealing and extension steps for 45 s at 56°C. We identified a sample as male if one or more replicates amplified for both X and Y chromosome markers, or as female if one or more replicates amplified, but only for the X chromosome. While results could not be conclusively evaluated for accuracy owing to lack of relevant necropsy data, we compared DNA sex identification results to predicted sex-specific patterns of hormones; for example, prolonged elevations of progesterone are expected to occur in females only.

### 2.3. Stable isotope analysis

Approximately 1 mg of baleen powder from each sampling location (i.e. every 1 or 2 cm along the longitudinal axis on each baleen plate) was transferred into tin capsules for isotopic analyses. Bulk *δ*^13^C and *δ*^15^N were measured using a Thermo FlashSmart elemental analyser coupled to a Thermo Finnigan Delta Plus XP continuous-flow isotope ratio mass spectrometer (Thermo Scientific, Bremen, Germany). Results are expressed in parts per thousand (‰) and delta notation (*δ*) using the equation: *δ*_sample_ = [*R*_sample_/*R*_standard_ − 1] × 1000, where *R*_sample_ and *R*_standard_ are the ^13^C/^12^C or ^15^N/^14^N ratios of the sample and the internationally used standards VPDB for carbon and AIR for nitrogen, respectively [44]. The isotopic reference materials used were supplied by the International Atomic Energy Agency (IAEA-N-1, *δ*^15^N = 0.4 ± 0.2‰; IAEA-CH-7, *δ*^13^C = −32.1 ± 0.05‰; IAEA-CH-3, *δ*^13^C = −24.7 ± 0.04‰) and the United States Geological Survey (USGS25, *δ*^13^C = −34.58 ± 0.06‰, *δ*^15^N = −0.94 ± 0.16‰; USGS40, *δ*^13^C = −26.3 ± 0.04‰, *δ*^15^N = −4.5 ± 0.1‰; USGS41, *δ*^13^C = +37.6 ± 0.04‰, *δ*^15^N = 47.6 ± 0.2‰). Internal standards were included with all samples as quality controls (purified methionine, Alfa Aesar, *δ*^13^C = −34.5 ± 0.06‰, *δ*^15^N = −0.9 ± 0.1‰; homogenized Chinook salmon muscle, NOAA Auke Bay Laboratories, *δ*^13^C = −19.2 ± 0.05‰, *δ*^15^N = 15.5 ± 0.1‰). The analytical precision, based on the standard laboratory replicas’ standard deviation, was 0.11‰ for *δ*^13^C and 0.12‰ for *δ*^15^N. All error data are s.d.

#### 2.3.1. Isotopic variation in baleen plates and isotopic niche widths

Statistical calculations on the stable isotope data were carried out with OriginLab 2025, a software for data analysis and graphing, and the R language [45]. The normal distribution of data was tested using the Shapiro–Wilk test. As latitudinal isotope gradients affect carbon as well as nitrogen isotopes of the whales’ food [46,47], we tested for relationships between *δ*^15^N and *δ*^13^C within a single baleen plate using Spearman's rank-correlation coefficients. Mann–Whitney tests were carried out to test the null hypothesis of whether two groups (female versus male) are not statistically different. Isotopic niches were determined using the Stable Isotope Bayesian Ellipses in R (SIBER) package [48]. This methodology uses bivariate ellipses using a Bayesian modelling approach to compare datasets with different sample sizes and ultimately isotope niche widths between and within communities [48]. The package provides estimates of the total area (TA), standard ellipse area (SEA) and sample size-corrected SEAs (SEAc). The calculation of standard ellipses incorporates 40% of all potentially eligible samples that would fall within the respective niches. The significance level was set at 0.05 for all analyses.

#### 2.3.2. Baleen growth determination

The isotope sequences represent a time series and can be treated as such, as baleen growth is continuous throughout the life of a baleen whale [3], and samples were taken sequentially at 1 or 2 cm intervals at the upper edge of all baleen plates (figure 2A). The whales' migratory behaviour through isotopically distinct components in water masses [49] is reflected in their baleen plates in the form of putative annual cycles of *δ*^13^C and/or *δ*^15^N [4,50,51]. In general, these oscillations were more pronounced in nitrogen data, and thus, in most cases, we relied on *δ*^15^N to estimate BGR. However, the carbon isotope data were used to estimate the BGR for three individuals. BGRs were determined using spectral analyses in PAST (PAleontological STatistics) v.4.17, a free software package released by the Natural History Museum of Oslo [52]. This analysis allows the calculation of peak frequencies to find significant periodicities in the isotope data, with period assumed to represent one calendar year. The spectral peak frequencies are given in 1/(*x*-unit), accordingly translated to *X*/cm.

**Figure 2.**
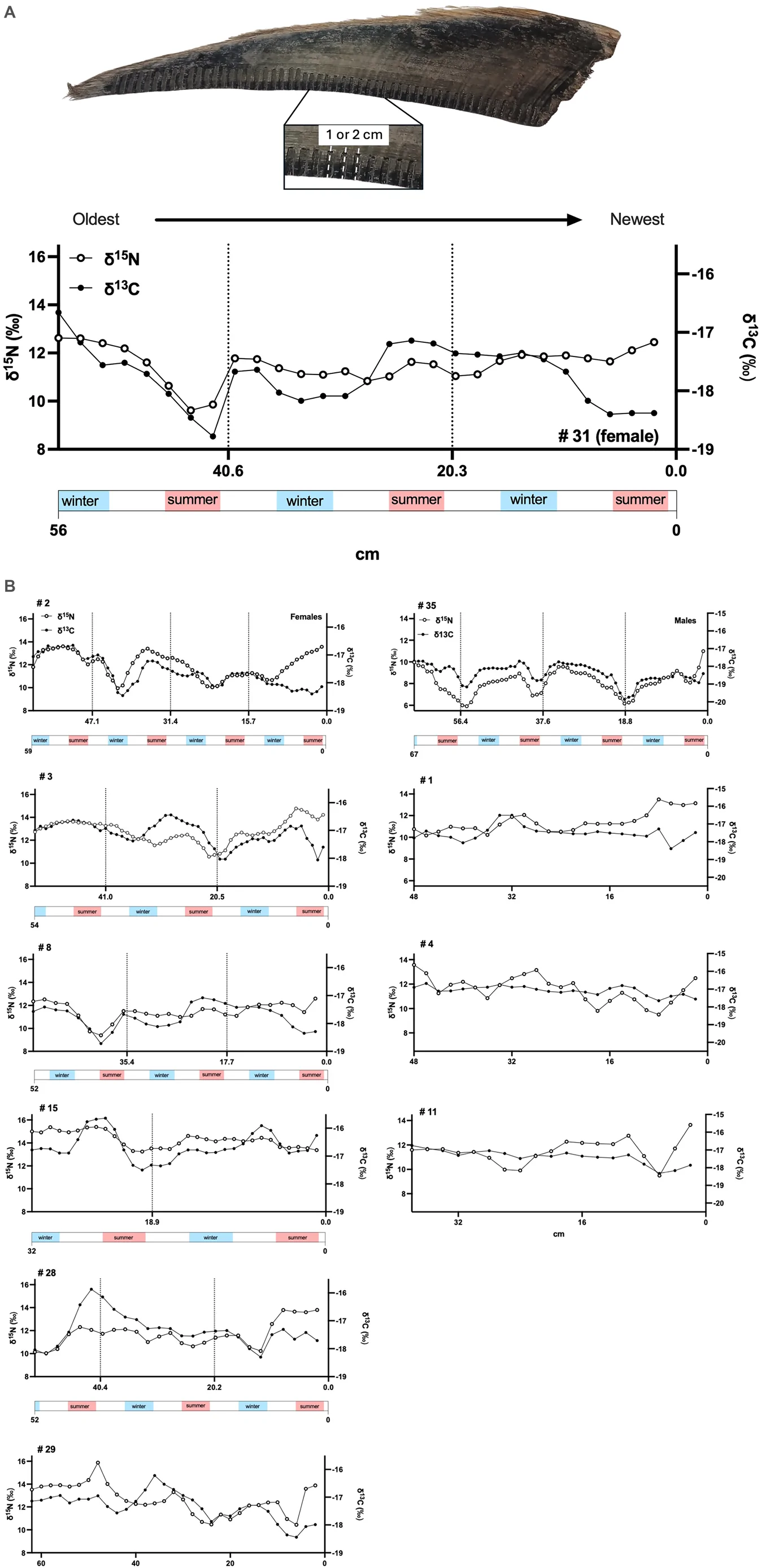
(A) Representative baleen plate showing the subsampling grooves (spaced at 1–2 cm intervals) and corresponding stable isotope profiles. Nitrogen (*δ*^15^N, open circles) and carbon (*δ*^13^C, solid circles) isotope values are presented along the length of the baleen plate, from oldest to most recent growth. Seasonal periods are indicated below the *x*-axis, with austral summer shown in salmon and winter in blue; autumn and spring are shown in white. Seasonal periods were reconstructed retrospectively from the estimated date of death (15 March 2015) using the individual baleen growth rate (BGR) of each whale. Meteorological seasons of the Southern Hemisphere were defined as follows: summer (1 December–28/29 February), autumn (1 March–31 May), winter (1 June–31 August) and spring (1 September–30 November). Calendar dates were converted to retrospective baleen positions (cm), allowing seasonal boundaries to be represented as continuous intervals along each baleen plate. (B) Stable isotope profiles of male and female individuals. Note that *δ*^15^N and *δ*^13^C are displayed on separate *y*-axes, and axis scales differ among individuals and between sexes. Baleen plates were subsampled at 2 cm intervals, except for individuals 02, 03, 15 and 35, which were sampled at 1 cm resolution. Baleen growth rate (BGR) was not determined for female 29 and males 1, 4 and 11.

### 2.4. Hormone analyses

#### 2.4.1. Hormone extraction

Steroid hormone extraction followed a protocol previously described by Hunt *et al.* [13,26,53] and optimized by Fernández Ajó *et al.* [30]. Powdered baleen subsamples (target mass 20.5 mg, acceptable range 20.5–21.0 mg, weighed to nearest 0.1 mg) were placed individually in 13 × 100 mm^2^ borosilicate glass tubes with 1.6 ml of 100% methanol, corresponding to an 80:1 ratio of ml of solvent to g of sample. The sample mixture was vortexed for 2 h at room temperature (Large Capacity Mixer, Glas-Col, Terre Haute, IN, USA; speed set to 40), centrifuged for 3 min at 4000*g* and the supernatant was transferred to a new 13 × 100 mm^2^ borosilicate glass tube and dried at 45°C for a minimum of 4 h in a sample evaporator (SpeedVac 121P, Thermo Fisher Scientific, Waltham, MA, USA) under vacuum. Dried samples were reconstituted in 0.50 ml of enzyme immunoassay (EIA) buffer (buffer ‘X065’, Arbor Assays, Ann Arbor, MI, USA), vortexed for 5 min, sonicated for 5 min, transferred to 1.5 ml cryovials with vapour-proof O-ring caps and stored at −80°C overnight. The resuspended sample was thawed and decanted into a new cryovial, thus removing any remaining sediment at the bottom. This was considered the ‘1:1’ (full-strength, neat) extract and was stored at −80°C until assay.

#### 2.4.2. Hormone assays

A commercial colorimetric EIA kit was used to measure the progesterone content of baleen extracts (Arbor Assays progesterone #K025, Ann Arbor, MI, USA) from 9 out of the 11 plates. The assay followed the manufacturer's protocol without modifications. The initial dilution for all assays was 1:2, with re-dilutions and reassay if results fell outside the range of the standard curve (i.e. very high progesterone). All samples of males were measured at a 1:2 dilution, while samples from pregnant females were diluted from 1:2 up to 1:128. Cortisol and corticosterone (#K003 and #K014, respectively, Arbor Assays progesterone #K025, Ann Arbor, MI, USA) were also measured at a 1:2 dilution; no further dilutions were necessary, as all values fell within the range of the standard curve. All three assays were previously validated for sei whale baleen extract [37].

Standard QA/QC for these assays in our laboratory includes assay of non-specific binding (NSB) wells, zeros, samples, standards and controls in duplicate, inspection of all assays for a normal range of optical densities, normal (low) NSB/zero ratio, good fit of standards to a four-parameter logistic curve and control results within expected bounds. Any samples with >10% coefficient of variation between duplicates (as measured from optical densities, i.e. to identify cases of pipetting error) were reassayed accordingly. Inter-assay and intra-assay precisions for all assays (as measured from interpolated doses derived from the average optical densities of pairs of wells, i.e. to assess the precision of final data) were all <10% (9.56% and 2.78% for progesterone, 6.24% and 2.11% for cortisol and 9.24% and 1.56% for corticosterone). For additional assay details, including sensitivity limits and cross-reactivities, see Hunt *et al.* [31,37]. Final data are expressed as ng of hormone per g of dried baleen powder.

#### 2.4.3. Determination of pregnancy length, calving interval and reproduction synchronicity

Progesterone peaks, that is putative pregnancies, were identified via a method commonly used for pregnancy diagnosis in mammalian wild life [54], namely, progressive rounds of elimination of any values higher than the mean ± 2 s.d. of all progesterone measurements in the same plate, until no more such outliers could be identified. The remaining samples define ‘baseline’ progesterone for that individual. The calculation of progesterone peaks was consistently performed using the original sampling interval of 4 cm to minimize bias, as areas with higher progesterone concentrations were proportionally more extensively tested. Pregnancy was inferred when an elevated and sustained progesterone concentration was identified for at least seven consecutive points. Intercalving interval was defined as the interval between successive births, comprising one gestation and the intervening non-pregnant period. The non-pregnant interval, defined here as elapsed time from the end of putative pregnancy to the beginning of the next (i.e. not including the subsequent gestation period), was defined as a region of baleen following a progesterone peak until the initiation of a subsequent peak. To estimate the duration of each interval, the length of the baleen separating progesterone peaks was divided by the annual BGR [17].

Reproductive synchrony across females was evaluated by examining the range of estimated dates of conception and birth of calves, based on the hypothetical date of death of all females, 15 March 2015—the midpoint between mid-February and mid-April 2015, the time period during which the mass stranding is thought to have occurred [1]. This estimated date of death was assigned to the proximal-most point of the plate, with more distal points assigned dates based on the BGR of that whale (from that individual's isotope data) and cm from the base of the plate. The estimated month of conception and the estimated month of birth were then compared across individuals.

Longitudinal profiles of cortisol and corticosterone concentrations were visually examined to identify periods of elevation across the baleen plates, with particular attention to patterns occurring during and after parturition. As even relatively low glucocorticoid peaks in baleen samples may be physiologically relevant, the ±2 s.d. method employed above for progesterone data was not used for the two glucocorticoids. Instead, we performed a simple exploratory visual assessment of relative highs and lows across each plate.

## 3. Results

### 3.1. Genetic sex determination

DNA was amplified from all samples, and all samples were successfully assigned as either male (*n* = 4) or female (*n* = 7; table 1). The sex of sei whale specimens corresponded to expected patterns in reproductive hormones (i.e. specimens identified as female had progesterone patterns suggestive of pregnancies; see below). All PCR and extraction blanks failed to amplify, indicating no contamination.

**Table 1.** Baleen length, stable isotope (SI) sampling interval, estimated growth rates and retrospective timelines for 11 sei whales (*Balaenoptera borealis*) stranded in central Chilean Patagonia between February and April 2015.

	sex	SI	baleen length (cm)	SI sampling interval (cm)	annual growth rate (cm)		mean annual growth rate (cm)	mean monthly growth rate (cm)	retrospective timeline (years)^a^		mean retrospective timeline (years)
					min	max			min	max	
whale 2	F	*δ*^15^N	59	1 and 2	15.2	16.2	15.7	1.31	3.6	3.9	3.7
whale 3	F	*δ*^15^N	54	1 and 2	19.4	21.5	20.5	1.70	2.5	2.8	2.7
whale 8	F	*δ*^13^C	52	2	15.4	20.0	17.7	1.47	2.6	3.4	2.9
whale 28	F	*δ*^15^N	52	2	18.2	22.2	20.2	1.68	2.3	2.9	2.6
whale 29	F	*δ*^15^N	62	2	ND	ND	ND	ND	ND	ND	ND
whale 31	F	*δ*^13^C	56	2	16.6	24.0	20.3	1.69	2.3	3.4	2.8
whale 15	F	*δ*^15^N	32	1 and 2	16.0	21.8	18.9	1.57	1.5	1.7	1.7
whale 4	M	ND	48	2	ND	ND	ND	ND	ND	ND	ND
whale 1	M	*δ*^15^N	50	2	ND	ND	ND	ND	ND	ND	ND
whale 35	M	*δ*^15^N	67	1 and 2	16.8	20.7	18.8	1.57	3.2	4.0	3.6
whale 11	M	ND	38	2	ND	ND	ND	ND	ND	ND	ND

*Note:* Growth rates and retrospective timelines were derived from *δ*^13^C or *δ*^15^N analyses. ND: not determinable.

^a^ Based on the min and max annual growth rate.

### 3.2. Isotopic variation and isotopic niche widths

All baleen specimens showed regular oscillations in their stable isotope data (figure 2B). These presumed annual cycles were generally more pronounced in the *δ*^15^N values than in the *δ*^13^C baleen values. The average *δ*^15^N range within a single baleen plate was 3.5‰ but reached up to 5.4‰ in whale 29. The average intra-individual *δ*^13^C range was 1.8‰. *δ*^15^N mean values ranged between 11.4‰ (whale 1) and 12.8‰ (whale 3), and *δ*^13^C mean values between −17.1‰ (whale 4) and −17.8‰ (whale 8). Out of all baleen specimens analysed, the baleen isotope values of one male individual (whale 35) exhibited the lowest *δ*^15^N as well as *δ*^13^C mean values, with 8.1‰ and −18.4‰, respectively (figure 2B). The highest baleen isotope values were observed in a female individual (sample ID: #15), which exhibited a *δ*^15^N mean value of 14.3‰ and a *δ*^13^C mean value of −16.7‰ (figure 2B).

Of 11 baleen plates, 6 showed a significant positive correlation between nitrogen and carbon isotope ratios internally (whale 2: *r_s_* = 0.49, *p* < 0.0001, *n* = 59; 04: *r_s_* = 0.47, *p* < 0.05, *n* = 24; 15: *r_s_* = 0.59, *p* < 0.0001, *n* = 32; 28: *r_s_* = 0.49, *p* < 0.05, *n* = 25; 29: *r_s_* = 0.42, *p* < 0.05, *n* = 31; 35: *r_s_* = 0.71, *p* < 0.0001), while 5 showed no such correlation (whale 1: *r_s_* = 0.05, *p* = 0.82, *n* = 25; 03: *r_s_* = 0.17, *p* = 0.21, *n* = 54; 08: *r_s_* = 0.32, *p* = 0.10, *n* = 26; 11: *r_s_* = 0.04, *p* = 0.86, *n* = 19; 31: *r_s_* = 0.30, *p* = 0.12, *n* = 28). Mann–Whitney tests showed that males had significantly lower *δ*^15^N and *δ*^13^C values than females (for *δ*^15^N: *U* = 2969.5, *z* = 9.5766, *p* < 0.0001; for *δ*^13^C: *U* = 6029.5, *z* = 4.8108, *p* < 0.0001).

SIBER analyses revealed that males (14.20‰^2^) exhibit a slightly higher TA than females (12.04‰^2^). The SEAs corrected for sample size (SEAc) are 2.60‰^2^ for male individuals and 1.94‰^2^ for female individuals (figure 3).

**Figure 3.**
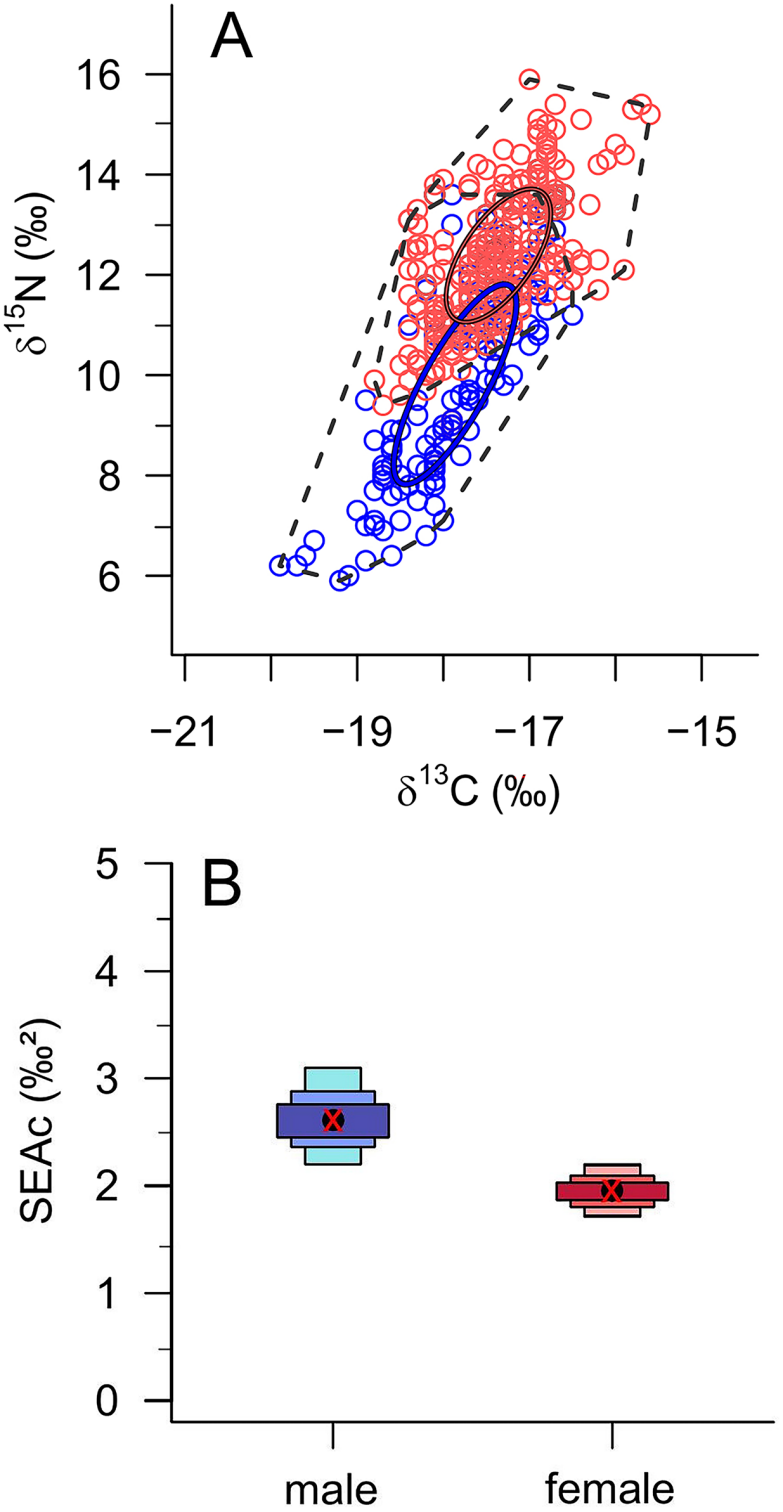
(A) Standard ellipse areas corrected for sample size (SEAc, solid ellipses) and convex hulls (dashed lines) of baleen plates from male (blue) and female (red) individuals. (B) Density plots of baleen values from male (blue) and female (red) individuals. Black dots indicate mode values for SEA-b, the Bayesian estimate for SEA. Density plots show credibility contours from the inside out at 50%, 75% and 95%. Red crosses represent SEA values corrected for sample size.

### 3.3. Isotope cycles and baleen growth rate

Baleen length varied from 32 to 62 cm in females and 38 to 67 cm in males. When applying spectral analyses to the C and N isotope records, significant cyclicity was evident in five of six female baleens. Three out of four male baleen plates showed no significant cyclicity. However, apparent cyclicity also varied with sampling interval: all four baleen plates sampled every 1 cm showed reliable stable isotope cyclicity, including one male (table 1). In contrast, four of seven baleen plates sampled at 2 cm intervals did not exhibit statistically significant stable isotope oscillation, both from males (table 1).

Stable isotope cycling also varied with the chemical element (C or N). Most of the whales presented significant cycles of *δ*^15^N (whales 2, 3, 15, 28 and 35), while whales 8 and 31 showed cyclicity only in *δ*^13^C. Whales 4 and 11 (both sampled only at 2 cm) did not exhibit cycling of either isotope. Whales 1 and 29 showed cyclicity but without statistical significance.

Average cycle length for each individual whale (i.e. average BGR) spanned 15.7–20.5 cm yr^−1^. The number of stable isotope cycles (putative years) varied from 1.7 to 3.7 years in females and from 3.2 to 4.0 years in the only male 35 in which cycles were detected (table 1). BGR could not be calculated for the three males that did not exhibit statistically significant isotope oscillations.

The shortest baleen (female 15) and the longest (male 35) only have stable isotope data. Both exhibited significant stable isotope oscillations, which enabled the determination of the baleen's growth rate (figure 1).

### 3.4. Hormone analyses

Progesterone was detected in all powder subsamples of all nine plates tested for this hormone (table 2). Sustained elevations in progesterone levels, that is putative pregnancies, were observed in all adult females. A bimodal progesterone peak was observed in all pregnant whales. The threshold of progesterone peaks varied from 350.8 ng g^−1^ in whale 28 to 556.5 ng g^−1^ in whale 8 (table 2). The highest progesterone concentrations of each presumably pregnant whale ranged from 985.6 ng g^−1^ in whale 3 to 4050.3 ng g^−1^ in whale 28.

**Table 2.** Baleen progesterone concentration and inferred reproductive parameters of nine sei whales (*Balaenoptera borealis*) stranded in central Chilean Patagonia, February–April 2015.

		progesterone (ng g^−1^)			pregnancy length (months)					
	sex	min	max	progesterone peak threshold (ng g^−1^)	min	max	mean pregnancy length (months)	initial month of pregnancy death in mid-Mar	last month of pregnancy death in mid-Mar	potential non-calving period (years)
whale 2	F	10.1	1379.8	480.0	10.4	11.1	10.70	Feb-13	Jan-14	≥1.7
whale 3	F	10.5	985.6	552.3	ND	ND	ND	ND	Jan-13	≥1.95
whale 8	F	8.3	1505.5	556.5	7.8	10.1	8.8	Jun-12	Mar-13	≥2.03
whale 28	F	17.9	4050.3	350.8	8.1	9.9	8.9	Jan-14	Oct-14	≥1.44
whale 29	F	15.5	1582.1	469.9	ND	ND	ND	ND	ND	ND
whale 31^a^	F	17.6	1181.7	403.2	6.5	9.4	7.7	Aug-12	Mar-13	>0.64
					7.0	10.1	8.3	Nov-13	Jul-14	>0.64
whale 4	M	9.9	56.1	ND	ND	ND	ND	ND	ND	ND
whale 1	M	6.6	35.1	ND	ND	ND	ND	ND	ND	ND
whale 11	M	14.1	113.6	ND	ND	ND	ND	ND	ND	ND

*Note:* Female 15 and male 35 are excluded here as their samples were not assayed for progesterone.

^a^ Two distinct pregnancy events were identified for whale 31 from the same baleen plate.

Mean pregnancy duration varied from 7.7 to 10.7 months (figure 4A–D,F). Two of the six females exhibited more than one progesterone elevation in their baleen plates. In whale 3, the first pregnancy was only partially recorded because its onset preceded the retrospective timeline, and a second progesterone increase could not be conclusively interpreted as pregnancy, fetal loss or ovarian activity because it differed from the hormonal profile observed in the other females (figure 4B). Based on the length of progesterone peaks and the BGR, the observed non-pregnant interval in whale 3 was 1.47 years. In contrast, whale 31 exhibited two complete pregnancies, enabling estimation of a 1.3-year intercalving interval. Conceptions occurred during winter (*n* = 2), summer (*n* = 2) and spring (*n* = 1), while births were recorded across all seasons, indicating no clear seasonal pattern.

**Figure 4.**
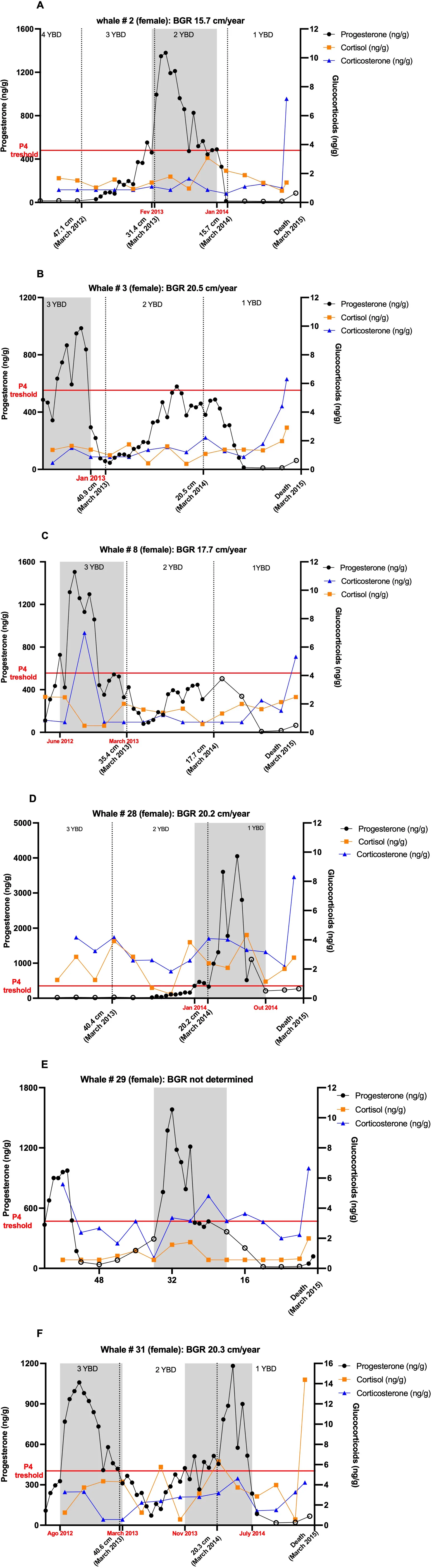
Baleen progesterone (black), cortisol (orange) and corticosterone (blue) records for female sei whales (A–F). Initiation and termination of pregnancy were retrospectively inferred based on baleen growth using the hypothetical date of synchronized death (mid-March 2015). Grey shaded areas indicate the inferred gestation period. Estimated times are expressed relative to the BGR, showing the number of years assessed retrospectively (YBD = year before death). P4 threshold indicated progesterone peaks in red horizontal lines. For progesterone, sampling points at 1 cm intervals are represented by black filled circles, whereas open circles represent sampling intervals of 2 or 4 cm.

Cortisol concentrations increased during the presumed pregnancy period in whales 2, 8, 28, 29 and 31 (figure 4A,C–F), with the highest values generally occurring during the latter portion of gestation. Elevated corticosterone concentrations were observed in whales 8, 28, 29 and 31 (figure 4C–F). In whales 8 and 31 (second gestation), corticosterone concentrations increased during the decline of progesterone following the period of highest progesterone values. Similar but less pronounced increases were observed in whales 28 and 29.

No clear glucocorticoid elevation was observed in whale 3 (figure 4B); however, glucocorticoid measurements were not available throughout the entire presumed pregnancy period.

Males exhibited no peaks in progesterone, with baseline concentrations varying from 6.6 to 113.6 ng g^−1^ (table 2; figure 5A–C). The progesterone concentration differed by an order of magnitude, more than 10 times, between presumably pregnant females and males. The most proximal subsample (1 cm) showed an apparent increase in progesterone concentration, a common occurrence in proximal-most baleen points termed the ‘moribund pattern’ by Evey *et al.* [55]. This elevation in hormones in proximal-most baleen may represent reduced clearance of hormones from circulation by liver and kidneys in a moribund animal; an alternative explanation is an artefact caused by circulating hormones in adjacent gum tissue [55].

**Figure 5.**
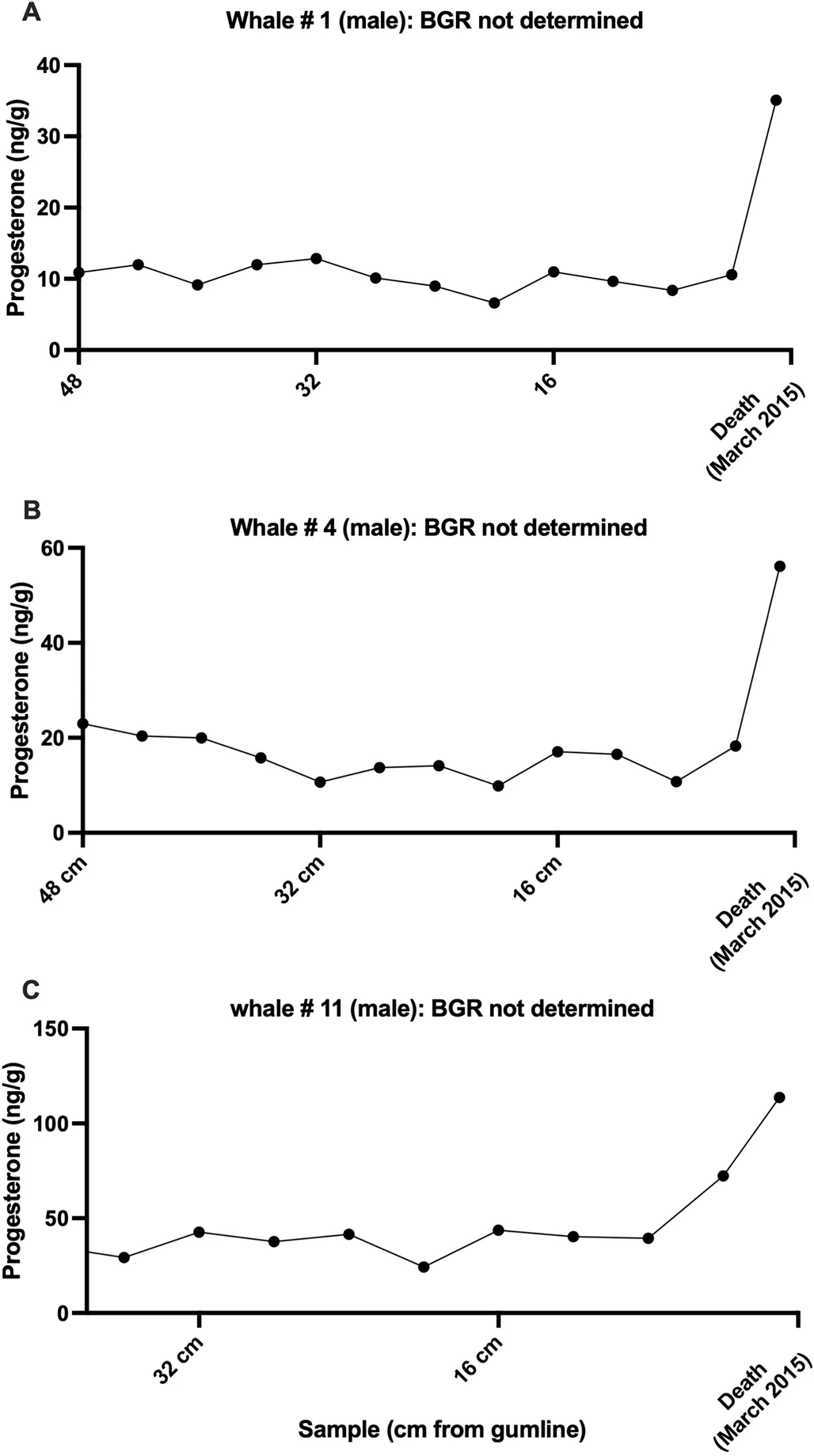
Baleen progesterone records for male sei whales (A–C).

## 4. Discussion

The collection and analysis of baleen plates from whales with unknown life histories can provide valuable insights into the biology and ecology of these species. Here, we demonstrate that integrating improved molecular techniques for sex determination with stable isotope and hormone analyses enabled the first comprehensive multi-year reconstruction of the life history of individual sei whales.

### 4.1. Sex identification from baleen

This study demonstrates the successful application of a refined DNA amplification protocol for sex identification from baleen in sei whales. Prior efforts have revealed that DNA does not always amplify from whale baleen using traditional endpoint PCR (unpublished data), even though that method works well for cetacean soft tissues (skin biopsy samples, stranded carcasses etc.) [56,57]. These prior failures may be owing to the low quality or quantity of DNA inherent to this sample type or may be owing to a methodological artefact, perhaps related to the unusual calcium-salt matrix of baleen. More recently, another modified forensic protocol employing organic solvents has also been successfully applied to extract DNA from the bones of sei whales stranded in the Golfo de Penas, Chile, for phylogeographic analyses [58]. Molecular sex determination from baleen whale material has previously been reported in archaeological samples [15]. Our results therefore expand the application of molecular sex identification to contemporary sei whale baleen, demonstrating its potential as an alternative approach for obtaining sex information when conventional morphological or field records are unavailable.

### 4.2. Stable isotope annual cycles

Some individuals presented reliable frequencies in the cyclic oscillations for both isotopes (*n* = 3). However, most individuals showed significant *δ*^15^N oscillations (*n* = 5). Similarly, oscillations were more evident in the *δ*^15^N and less pronounced in the *δ*^13^C in the baleen plates of six sei whales collected during the same stranding event on the Chilean coast [38]. Such variations are related to the latitudinal gradients in the N and C isotope values of nitrate [46] and dissolved inorganic carbon, and consequently, also of particulate organic matter [47].

In the Southern Hemisphere, a strong isotope gradient is particularly evident in latitudes between 40 and 60°S for both isotopes [49,59]. The *δ*^15^N values of nitrate, for instance, decrease by about 3‰ along a latitudinal gradient in the eastern Pacific within a 20° latitude range, a phenomenon caused by isotopic fractionation resulting from different nitrate use by phytoplankton [38,46]. Accordingly, the *δ*^15^N and *δ*^13^C gradients are reflected in the food sources along the whales’ migratory routes and are ultimately manifested in the isotopic profiles of their baleens. These spatial gradients are also depicted in models known as isoscapes, which map the isotopic composition of nitrogen and carbon and are used to track biogeochemical processes, nutrient uptake by phytoplankton and, not least, the migration patterns of various animal species [60].

Three of the four studied males (1, 4 and 11) did not present consistent cyclicity of any of the isotopes, irrespective of the length of the baleen. Though this may be an artefact of sampling interval, interindividual isotope variability of baleen could be attributed to different migration patterns of individual animals as some individuals stay longer in a particular feeding area [4,38,61–63]. Accordingly, irregular movements between different regions, or even year-round residence in the same area or latitude, would lead to less pronounced seasonal *δ*^15^N variations in the baleen [4,63]. Such behaviour would thus influence the estimates of growth rates [4] and could be the reason for the lack of isotope periodicity in these males (1, 4 and 11). However, this could also be owing to their overall baleen lengths, which were among the shortest in this study. In contrast, most females exhibited cyclicity in the nitrogen isotope ratio, indicating both a high fidelity to foraging zones in the Southeast Pacific across years and a certain latitudinal migratory pattern along an isotope gradient [38]. However, SIBER analyses showed that male individuals tend to have larger isotopic niche widths than female individuals (figure 3). In particular, male individual 35 showed overall lower *δ*^15^N values and therefore probably spent more time in waters with a lower isotope signature at more southerly latitudes [49].

Consistent with this pattern, stable isotope records from fin whale baleen plates (*δ*^15^N and *δ*^13^C) demonstrate that climate variability drives shifts in diet and migration, with *δ*^15^N values reflecting changes in trophic level and *δ*^13^C values indicating variation in habitat use and latitudinal movements [64]. Accordingly, the analysis of stable isotopes in the baleen whale tissues can serve as an indicator for tracking how changing climatic conditions and marine ecosystem dynamics can affect feeding ecology. Continuous isotope profiles reconstructed from the whales’ earplugs, for instance, revealed environmental and behavioural changes over several decades [65]. Similarly, carbon and nitrogen isotope ratios obtained from biopsies of humpback whales’ skin and blubber tissues showed a high degree of foraging plasticity, which resulted in adjusting their dominant prey sources in response to local habitat conditions, climatic anomalies and shifts in the productivity of nearshore ecosystems [66]. In Antarctic waters, rapid regional warming correlated with changing prey availability and flexibility in the foraging behaviour of various balaenopterid species [67]. Whales can therefore serve as sensitive indicators of long-term, climate-driven oceanographic changes.

In the 4 years preceding the 2015 mass stranding, corresponding to the maximum retrospective period covered in the present study, no abnormal isotopic oscillations were detected that would suggest changes in migratory patterns and/or feeding frequency of sei whales. Since 1970, three major ENSO events (1982–1983, 1997–1998 and 2015–2016) have been recorded, all associated with increases in sea surface temperature, increased upwelling and the occurrence of HABs in the Chilean Patagonian fjords [1,68]. These findings suggest that the 2015 event may not have been preceded by detectable long-term ecological or behavioural shifts in sei whales but rather probably resulted from an acute, episodic environmental perturbation that rapidly propagated through the food web—a phenomenon that is, however, difficult to detect based on N and C baleen isotope values. The incorporation time of elements into keratin tissue varies among the specific elements, for example 78 days for sulfur and only a few days for carbon [64]. Short-term environmental events, such as HABs, may have been too acute to be detectable in the isotopic signature of the baleens. The origin of HABS is related to positive phases of climatic phenomena such as ENSO and the Southern Annular Mode, but local factors also play an important role [68]. Since an abnormal increase in *δ*^15^N values is not observed in the most recent sections of all baleens, migratory and feeding habits are the more likely cause for isotope cyclicity than local oceanographic changes.

Regarding potential food sources, Reiss *et al.* [38] discussed possible prey items from the broader area for various sei whale individuals that had stranded in the same region of the Patagonian fjords as part of the same mass mortality event. Based on the mean values of the stable C and N isotopes of the primary prey spectrum (euphausiids, amphipods and copepods) [37] and assuming a trophic enrichment factor of +2.8‰ for *δ*^15^N and +2.3‰ for *δ*^13^C [66], the results for most individuals indicate a strong overlap with Patagonian food sources, similar to the previously published baleen data [38]. Given the fact that no hormone analyses were performed in Reiss *et al.* [38], it is interesting to note that one male individual had baleen values that fell completely outside the range that can be explained by Patagonian food sources. This suggests that at least male individual 35 was probably not present in the Patagonian fjords during the formation of his baleen but only at the time of his death (figure 6).

**Figure 6.**
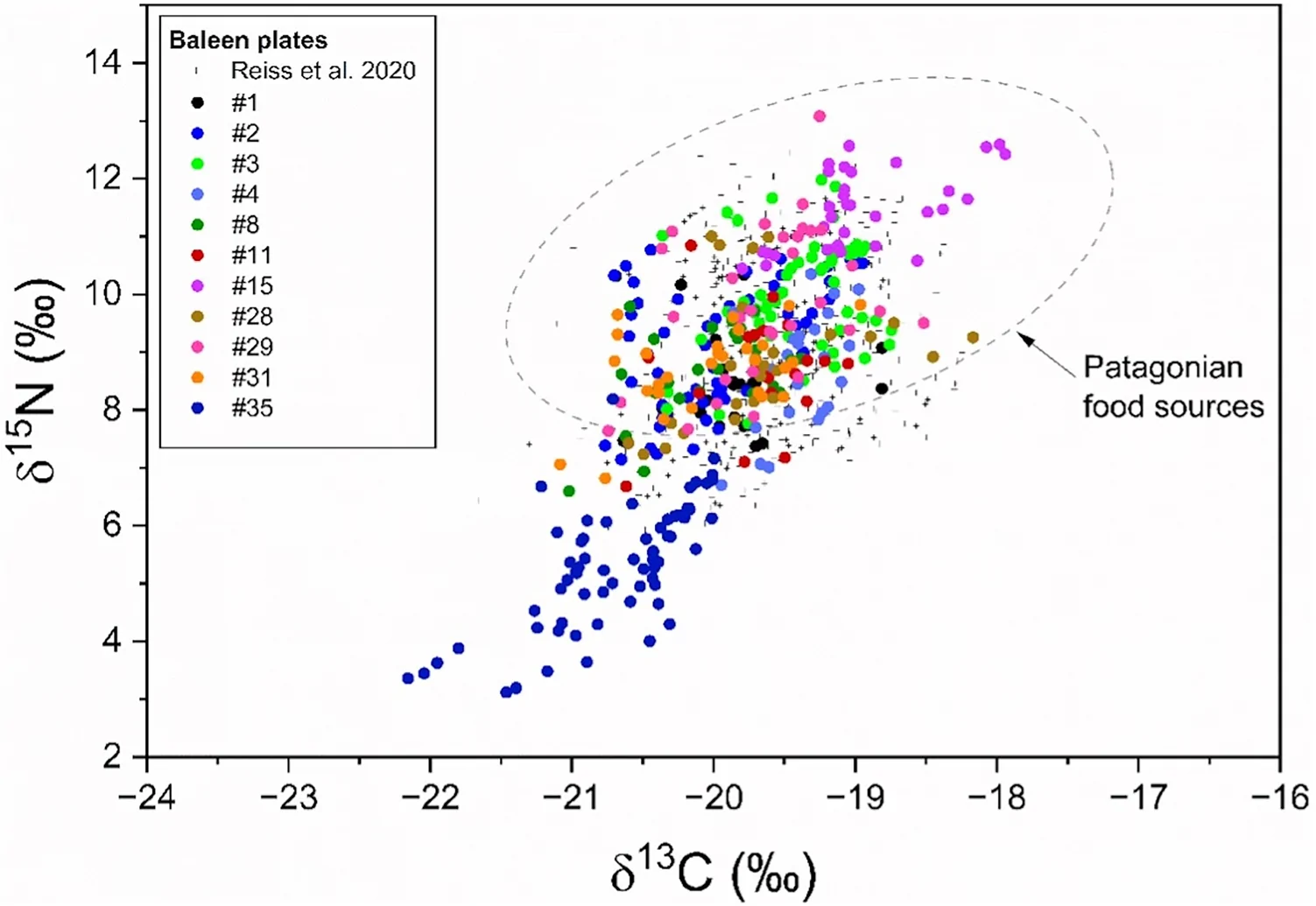
*δ*^15^N and *δ*^13^C values for baleen plates (*n* = 11) from this study (filled circles) and baleen plates (*n* = 7) from Reiss *et al.* [38] (black crosses), corrected for trophic fractionation (*δ*^13^C = 2.3‰ and *δ*^15^N = 2.8‰, according to Borrell *et al.* [69]). The dashed area represents the primary food sources in Patagonian fjords, as reported by Reiss *et al.* [38].

However, the overlap of male isotope niches with those of females (figure 3) indicates that they coexisted with female individuals at least at other times, such as during mating. In sei whale populations off the west coast of South Africa, pregnant and/or lactating females with calves were also found to join sexually mature males during the southern migration between August and October, that is austral winter to spring [70].

### 4.3. Baleen growth rate

Based on statistically significant stable isotope oscillations, the mean annual BGR was 18.9 ± 1.7 cm yr^−1^ (range: 15.7–20.5 cm yr^−1^), extending the upper range of previously reported estimates for sei whales [38]. This is broadly similar to BGRs reported for adults of most other mysticete species (blue, fin, humpback, Bryde's, bowhead, North Atlantic right whales) [4,13,22,25,51,64,71]; however, slower compared to BGR in minke whales, which are among the smallest mysticetes [72,73]. The greatest influence on BGR in mysticetes appears to be age (rather than species), with the BGR of calves often three times higher than that of adults, progressively slowing through juvenile and subadult years to approach a slow and asymptotic (little further change) BGR in the oldest adults [71,74]. BGRs can be further modulated by seasonal changes within annual feeding cycles, often slowing during presumed fasting periods (e.g. migration) and accelerating during feeding [71,75]. Another reason for the variability in BGR may also be related to the different growth rates at different positions of the baleen plates within the jaw, especially in calves [76]. *δ*^15^N values were preferred over *δ*^13^C values for detecting cycles of sei whales because of their more significant fluctuations and generally stronger signal owing to a greater trophic enrichment. However, *δ*^13^C values have also been used to determine BGRs of bowhead whales (*B. mysticetus*) [3,14,74] and southern right whales (*Eubalaena australis*) [77].

Validated protocols for sampling archival keratinous tissues, such as hair, feathers, vibrissae and baleen, have been widely applied to investigate ecological and physiological traits across species [37,78,79]. In baleen, growth rate can strongly influence the resolution and interpretation of stable isotope, hormone and molecular data, depending on the sampling scale. Generally, increased spacing between subsamples reduces the statistical power to detect potential time-series heterogeneity across the baleen plate, thereby diminishing both the sensitivity and precision of ecological and physiological inferences. Additionally, wide sampling intervals could, in theory, completely miss certain short-term events, for example acute stressors. In the present study, all baleen samples collected at 1 cm intervals exhibited consistent and reliable stable isotope cyclicity, including a female individual with a 32 cm long baleen plate. Conversely, four of the seven baleen plates sampled at 2 cm intervals did not exhibit significant stable isotope oscillations, including three males and one female (table 1). Although baleens sampled in lower resolution do not show significant changes in their average BGR, we recommend prioritizing higher-resolution measurements to ensure that all cycles are reliably detected and to increase the statistical significance.

The number of years represented by a given baleen plate is subject to both the baleen plate's length and the whale's BGR (in turn affected by whale age). For instance, balaenid whales (right whales and bowhead) possess the longest baleen plates, not only relative to body mass but also in absolute length, with lengths sometimes exceeding 3 m in adult bowhead whales [23]. Regular oscillations in stable isotope ratios, interpreted as annual cycles within these exceptionally long plates, have enabled the reconstruction of up to 22 years of life history in a single individual [27]. In sei whales, stable isotope cyclicity revealed that baleen plates preserved between 1.7 years (in the shortest plate) and up to 3.7 years of stable isotope data. The quantity and resolution of information retrievable within such time frames ultimately depend on the species-specific biological and physiological characteristics.

### 4.4. Progesterone and pregnancy

Our study revealed sustained progesterone elevations in sei whale baleen, suggestive of pregnancies, allowing for estimation of gestation length as well as intercalving and non-pregnant intervals. Based on the mathematical identification of progesterone peaks/elevations, the calculation of BGRs and the duration of these peaks in months, we estimated the mean gestation period of sei whales to be between 7.7 and 10.7 months, aligning with previously published estimates [36,80]. This is in contrast to balaenid whales (Balaenidae; bowhead and right whales), in which baleen progesterone patterns consistently indicate a much longer gestation length (>15 months) than had previously been estimated [13,17,81–83], possibly explaining why 2-year calving intervals are unknown in the balaenids. In contrast, the gestation duration of rorquals (Balaenopteridae), such as the sei whale, may indeed fit within one calendar year [16], enabling a hypothetical 2-year calving interval for females in good condition.

Most pregnant whales exhibited a bimodal pattern of progesterone elevation, for example an early progesterone peak, a dip (but still above baseline) and then a later progesterone peak within the same pregnancy (e.g. whale 28 shown in figure 4D). This pattern has also been reported in other mysticetes such as bowhead whales [17] and southern right whales [83], but not in odontocetes [84,85]. Possible explanations for this bimodal peak include delayed implantation (e.g. the second peak may represent the time of implantation) [17,86–88] or a polyestrous pattern of more than one ovulation per year (e.g. an initial luteal phase that did not result in pregnancy) [17]. However, such phenomena are relatively uncommon in migratory species with synchronous seasonal breeding, as in such cases, females are usually monoestrous (ovulate once and mate successfully). A third explanation is a transition in progesterone production from the corpus luteum to the placenta, a common phenomenon in many mammals [89,90]. We hypothesize that the dip between the two peaks represents the ‘handover’ of progesterone production from corpus luteum to placenta, as seen in several other mammalian orders [91].

Among reproductive parameters in whales, calving intervals provide critical insights into population demographics and life-history traits [92]. Intercalving interval can vary within and among individuals with resource availability, environmental conditions and health status [17]. Determination of calving interval is particularly relevant for species that were heavily exploited in the past and have not yet recovered, such as the sei whale. Historically, calving intervals have been estimated via photo-identification of known individuals [93–95]. While effective, this approach is limited to species that exhibit strong calving site fidelity, with consistent sighting efforts paired with catalogue information of known individuals. Analysis of progesterone patterns from baleen, however, can often enable estimation of calving intervals even in individuals without sighting histories [16,17]. Previous estimates of sei whale calving intervals of 2–4 years were inferred from pregnancy rates obtained from females killed during commercial whaling [35,80,96,97]. In our study, one female had two complete gestations recorded in the baleen, enabling an estimation of an intercalving interval of 16 months. In another female, a non-pregnant interval of 1.47 years was estimated based on the spacing between progesterone peaks and the estimated BGR. However, this estimate should be interpreted with caution because the first pregnancy was only partially recorded, and a subsequent progesterone elevation could not be conclusively interpreted as pregnancy, fetal loss or ovarian activity (whale 3; figure 4E). All remaining females exhibited only a single pregnancy episode in baleen records spanning a mean retrospective period of 2.9 years (range: 1.7–3.7 years). Nonetheless, our data demonstrate that even the relatively short baleen plates of sei whales can preserve sufficient reproductive information to estimate both non-pregnant and intercalving intervals.

### 4.5. Reproductive seasonality and synchrony

Most mysticete whales exhibit marked seasonality in reproduction, migrating from subtropical calving areas to circumpolar feeding grounds during the austral summer and spring, with calving often restricted to a relatively narrow 2–4 month time window [33,34,38]. Previous studies based on sei whales taken during commercial whaling suggested that, in the Southern Hemisphere, conception occurs over a relatively broad temporal window, with a peak during June and July, despite the absence of clear seasonal cycles in spermatogenesis [97,98]. In our study, conceptions occurred during the austral summer (*n* = 2), winter (*n* = 2) and spring (*n* = 1), whereas births were distributed across all seasons. Although the limited sample size precludes robust conclusions regarding reproductive seasonality, our findings are consistent with an extended breeding period rather than a tightly synchronized reproductive cycle. These findings suggest a degree of plasticity in the timing of sei whale calving, possibly related to the less predictable seasonal movements of sei whales compared to other cetaceans [97,99,100]. For example, sei whale summer feeding is notably unpredictable, often characterized by a sudden influx into an area, followed by rapid disappearance and subsequent absence for several years [100,101]. However, the calendar dates estimated for conception and parturition should be interpreted with caution, as they were calculated based on the assumed date of death of each animal, and the exact timing of death may have varied among individuals. If some animals died earlier or later during the stranding event, this variation could have introduced additional uncertainty into the estimated calendar dates and may have influenced the apparent seasonal distribution of pregnancy initiation and completion. Therefore, these calendar estimates, including their assignment to specific seasons, should be considered approximate.

### 4.6. Glucocorticoids

Finally, glucocorticoid data provided information regarding late gestation and potential lactation. Parturition in mammals involves a complex interplay of neuroendocrine mechanisms, which are highly conserved across most species [102]. Generally, fetuses make a significant endocrine contribution during late gestation through the release of glucocorticoids from their adrenal glands, which play a pivotal role in initiating parturition [91]. In captive odontocetes, increases in circulating cortisol and fecal metabolites have been documented during pregnancy, particularly in late pregnancy before parturition [103,104]. Discrete sampling of blubber and faeces has also revealed a relationship between pregnancy, characterized by elevated progesterone and increased concentrations of cortisol and glucocorticoid metabolites, indicating that this physiological phenomenon occurs in baleen whales as well [105,106]. More recently, retrospective hormone analyses of baleen from North Atlantic right whales and bowhead whales have demonstrated marked elevations in corticosterone at the end of the pregnancy period [17,31]. In the present study, discrete elevations of one or both glucocorticoids were consistently observed during mid- to late pregnancy, generally coinciding with declining progesterone concentrations. These patterns were evident in all but one female and suggest that glucocorticoids may provide information on physiological transitions associated with late pregnancy, parturition, initiation of lactation or a combination of these processes. Consequently, glucocorticoid profiles recorded in baleen may prove useful for distinguishing successful from unsuccessful pregnancies. Further exploration of this possibility would benefit from higher-resolution baleen sampling, longitudinal sighting data confirming calving success and lactation, and the identification of additional biomarkers associated with reproductive state.

## 5. Conclusions

Our study demonstrates that sex determination from baleen is feasible in sei whales and that a combination of retrospective analyses of stable isotopes and hormones can provide multi-year records of reproductive cycles. Sustained elevations of progesterone enabled the inference of gestation length, intercalving and non-pregnant intervals, while stable isotope niche modelling revealed broader feeding niche widths in males compared to females, but also differences in the absolute range of the values. On average, males had lower isotope values than females, indicating partly different migration routes and seasonal feeding habitats. Despite their cosmopolitan distribution, basic reproductive parameters of sei whales remain poorly understood owing to their elusive behaviour and deep-water habitat. Mass strandings, although tragic, provide rare opportunities to reconstruct reproductive histories and generate demographic parameters critical for assessing population resilience under historical exploitation, contemporary anthropogenic threats and climate-driven oceanographic change. Our findings close long-standing gaps in sei whale biology, a species that remains poorly understood despite its endangered status and global distribution. Future research should aim to expand sample sizes across different geographic regions and integrate baleen hormone profiles with longitudinal studies in living populations to validate pregnancy outcomes, calving intervals and potential reproductive synchrony. By combining molecular, isotopic and hormonal tools, baleen archives can yield crucial insights into the reproductive biology and ecology of sei whales, thereby filling key data gaps essential for informed conservation and management of this endangered species.

## Supplementary Material



## Data Availability

The dataset supporting this study has been deposited in the Zenodo repository [107]. Supplementary material is available online [108].
